# Awareness, offer, and use of psychosocial services by breast cancer survivors in Germany: a prospective multi-centre study

**DOI:** 10.1007/s00404-022-06665-3

**Published:** 2022-07-15

**Authors:** Susanne Singer, Wolfgang Janni, Thorsten Kühn, Felix Flock, Ricardo Felberbaum, Lukas Schwentner, Elena Leinert, Achim Wöckel, Tanja Schlaiß, for the BRENDA study group

**Affiliations:** 1 Division of Epidemiology and Health Services Research, Institute of Medical Biostatistics, Epidemiology and Informatics, University Medical Centre, Johannes Gutenberg University, Obere Zahlbacher Straße 69, 55131 Mainz, Germany; 2 Department of Gynaecology and Obstetrics, University of Ulm, Ulm, Germany; 3 Department of Gynaecology and Obstetrics, Hospital Esslingen, Memmingen, Germany; 4 Department of Gynaecology and Obstetrics, Hospital Memmingen, Memmingen, Germany; 5 Department of Gynaecology and Obstetrics, Hospital Kempten, Kempten, Germany; 6 Department of Gynaecology and Obstetrics, University of Würzburg, Würzburg, Germany; 7 University Cancer Centre, Mainz, Germany

**Keywords:** Breast neoplasms, Distress, Financial problems, Prospective studies, Supportive care, Psycho-oncology

## Abstract

**Purpose:**

This study examined the pattern of psychosocial care in breast cancer survivors.

**Methods:**

In a prospective study with measurements before surgery, 1 month, 8 months, and 5 years thereafter, we examined the proportion of breast cancer survivors who were aware about, had been offered and received various types of psychosocial services from psychologists, social workers, doctors, self-help groups etc. The degree of helpfulness per service among users was ascertained with Likert scales. Determinants of awareness, offer and use were investigated using binary logistic regression analyses. How the services are inter-related was tested with principal component analyses.

**Results:**

Among 456 breast cancer survivors who participated until 5 years, psychological services were known by 91%, offered to 68%, and used by 55% of patients. Social services were known by 86%, offered to 65%, and used by 51%. Women ≥ 65 years were less likely to be informed about (odds ratio (OR) 0.2) and get offers for psychosocial services (OR 0.4 for social and 0.5 for psychological services) than women < 65 years. The services rated most helpful were social services in the hospital, psychological counselling by a consultant and psychotherapy in private practices.

**Conclusion:**

These findings underline the importance of psychosocial support by physicians in addition to the "professional" mental health and social care providers. They also show that elderly women in need for support might be in danger of not being well-informed about the services available.

**Supplementary Information:**

The online version contains supplementary material available at 10.1007/s00404-022-06665-3.

## What does this study add to the clinical work

**Table:** 

Psychosocial support by non-mental health professionals (i.e., oncologists, general practitioners etc.) is considered to be very helpful by breast cancer survivors, yet not many of them receive it. It should therefore be offered in addition to the psychosocial services by mental health care professionals	

## Introduction

Psychosocial support for cancer patients should be an integral part of multi-disciplinary cancer care whenever there is a need for it. This goal is defined in the National Cancer Plan in Germany [1] as well as in many other countries [2]. But how many patients indeed receive such support? How many are even aware of it?

For the *inpatient sector*, we are able to answer the first of these questions. In cancer centres certified by the German Cancer Society, about 37% of all patients receive at least one consultation with a psycho-oncologist [3] and 75% with a social worker [4]. For hospitals that are not certified, no such data are available. According to a representative study with more than 6000 cancer survivors, 9% received at least one consultation with a psycho-oncologist in the hospital [5].

In Germany, every cancer patient has access to rehabilitation free of charge for 3 to 4 weeks. The costs are covered by the pension insurance companies. Patients must be admitted to the rehabilitation clinic (outpatient or inpatient) within two weeks after the end of the primary oncological treatment. Most cancer patients use inpatient rehabilitation clinics. There, each patient should receive at least one individual consultation with a psychologist plus group sessions [6–9].

In the *outpatient setting*, psycho-oncological care is primarily delivered by cancer counselling centres and in private psychotherapy practices [10–13]. Data on care provision in this setting are sparse, but it seems that about 10% of all cancer patients receive such support [5, 14, 15]. In a study with 927 breast cancer patients, 11% said 40 weeks after the discharge from the hospital they had experienced a need for psychological care and had also received it, whereas 6% experienced a need without getting such care and 76% reported having no need [16].

Little is known about psychosocial care by oncologists in private practice and self-help groups, both being valuable sources of emotional support [17]. Self-help groups differ considerably in their ability to attract members. Only 4% of lung cancer patients reported having ever visited a self-help group [18] whereas 23% of patients after total laryngectomy said they did so regularly [19].

It is important to differentiate carefully between the various health care providers when researching support options because the support they offer differs. In our view, nutritionists, for example, are very important to meet nutritional information needs, but they cannot provide psychotherapeutic help (and vice versa). In some studies investigating care provision, the different groups of carers were unfortunately combined quite broadly, which makes it difficult to estimate to what extent care needs are met [20, 21].

With our study, we aimed at defining the proportion of breast cancer survivors who are aware of and receive support by different health care providers. In detail, we asked, what is the proportion of breast cancer survivors who are aware of, have been offered, and have received:

- *Professional psychosocial support services* (i.e., psycho-oncological consultation in the hospital, social services in the hospital, cancer counselling centres, and psychotherapists in private practice)?
- *Basic psychosocial support services* (i.e., psychosocial counselling by general practitioner or consultant, general counselling centres, pastoral care)?
- Support from *self-help groups*?

We were also interested in the extent to which users experience the services as helpful and how the various services are inter-related. Finally, we investigated which groups of patients are aware of, being offered, and are receiving professional services.

Our assumptions were that a) it would be good if all patients knew about the services to an equal extent, i.e., independent of age, education, etc., and b) it would be good if the offer depends on the level of distress, not on demographic or socio-economic characteristics.

## Methods

### Study design

In a prospective multi-centre cohort study, patients with primary breast cancer were sampled consecutively [22, 23]. Participants were approached four times: before surgery (*t*1), 1 month after surgery (*t*2), after completion of adjuvant chemo-/radiotherapy (*t*3), and 5 years after surgery (*t*4).

### Instruments

*Distress* was measured using the short form of the Patient Health Questionnaire (PHQ-9) [24].

*Financial problems* were obtained with the respective subscale of the European Organisation for Research and Treatment of Cancer Quality of Life Questionnaire Core Instrument (EORTC QLQ-C30) [25] and its thresholds for clinical importance defined by Giesinger [26].

*Awareness and use of psychosocial care* were ascertained at *t*4 for a variety of services with a questionnaire developed by our group in several studies [27–29]. The instrument was in use in patients with cancer of the lung [27, 28], brain [30], and various sites including breast [29].

The services we asked about were:

- In the hospital: psychological consultation-liaison services, social services, pastoral services.
- In the inpatient rehabilitation clinic: individual consultations with psychologist, group sessions with psychologist, social services.
- In the outpatient setting: psychological services at cancer counselling centre, social services at cancer counselling centre, generic counselling centres, psychotherapists in private practice, psychological consultation by general practitioner, psychological consultation by consultant, self-help groups, pastoral services.

For each specific service, we asked whether patients knew that it exists, whether they had received an offer to use it, whether they had used it, and, how helpful it was from their point of view if they had used it (from very much to not at all on a 4-point Likert scale). If a person had not used social or psychological services, we asked for the reason of that decision.

*Clinical data* such as tumour stage, HER2-status, and treatment receipt were obtained from the medical records by trained data managers.

*Demographic data* such as age, education, income, migration history, and employment status were provided by the patient. Equivalent income was calculated based on the household net income divided by the number of adults and children in the household [31]. Participants were defined as "with migration history" if they were born outside of Germany and/or if they had a non-German citizenship and/or non-German nationality.

### Statistical analysis

Main outcomes of interest were awareness, offer, and use of psychosocial care. If a patient indicated she had used a certain service, missing values in the corresponding "awareness item" were replaced with "is aware of it" because one cannot use a service without being aware of it. Similarly, if a patient indicated she had been offered this particular service, missing values in the "awareness item" were replaced with "is aware of it". We did not replace missing information in the "offer item" even if a patient said she had used these services because it is possible that she had searched for it herself without being advised to use it.

Multivariate logistic regressions were performed to identify predicting factors for awareness, offer, and use of professional psychosocial care. We combined the services as follows:

- All types of professional *psychological support*, i.e. by psychotherapists and psycho-oncologists (combining inpatient and outpatient setting)
- All types of professional *social work support* (combining social services from inpatient and outpatient setting)

The following variables were considered to be potential predictors and were included in all models: age, education, income, migration history, risk for cancer progression (according to St. Gallen criteria [32]), and distress (for psychological services) or financial problems (for social services). We hypothesised that distress and financial problems are both related to psychosocial care, whereas all other variables should not be related to it.

The interrelation of services was investigated using Principal Component Analysis.

Statistical analyses were performed using STATA 12 (StataCorp 2011, College Station, TX: StataCorp LP).

## Results

### Sample

Of the 759 patients who participated at baseline [33], 60 had died by *t*4 (8%), 101 (13%) declined to participate again, one had moved to an unknown address, and 141 (19%) were not contacted due to budget restrictions, resulting in 456 participating survivors at 5 years after diagnosis (60%). Participants were on average younger than those who declined (+7 years) and those who died (+8 years). The participants' characteristics are displayed in Table 1.

**Table 1:** Respondents’ demographic and clinical characteristics at t4 (5 years after diagnosis)

	*N*	%
Age in years		
< 40	8	2%
40–49	50	11%
50–59	131	29%
60–69	123	27%
70–79	115	25%
80 +	19	4%
Education in years		
Unknown	10	2%
< 10	195	43%
≥ 10	256	56%
Unknown	5	1%
Current income in euros per person per month (equivalence income)		
< 500	18	4%
500 to 999	87	19%
1000 to 1499	102	22%
> 1500	186	41%
Unknown	63	14%
Immigrant		
No	387	85%
Yes	64	14%
Unknown	5	1%
Psychiatric comorbidity		
No	361	79%
Yes	89	20%
Unknown	6	1%
Locally advanced disease		
No	209	46%
Yes	247	54%
Surgical treatment		
Breast conserving	392	86%
Mastectomy	64	14%
Radiotherapy		
No	40	9%
Yes	416	91%
Chemotherapy		
No	247	54%
Yes	209	46%
Endocrine therapy		
No	84	18%
Yes	372	82%

### Awareness, offer, and uptake of services

Psychosocial services in the hospital and in the inpatient rehabilitation clinic were *known* by about three quarter of the survivors (Table 2). Services in the outpatient setting were known by about half of the patients. The lowest percentage is found in "Psychological counselling by general practitioners", where only 44% of all respondents were aware of this possibility.

**Table 2:** Awareness, offer, and uptake of psychosocial services by breast cancer survivors and the perceived helpfulness

	Percentage			If used: how helpful
	Aware of it	Offered	Used it	(0 = not at all, 3 = very much)
Hospital (*n* = 456)				
Psychological consultation-liaison services	75%	48%	21%	1.99
Social services	78%	55%	41%	2.40
Pastoral services	65%	35%	11%	2.02
Inpatient rehabilitation clinic (*n* = 242)				
Consultation with psychologist	81%	57%	54%	1.93
Group sessions with psychologist	79%	50%	48%	1.75
Social services	74%	48%	41%	2.12
Outpatient setting (*n* = 456)				
Cancer counselling centre: psychological services	68%	33%	9%	1.98
Cancer counselling centre: social services	58%	26%	9%	2.26
Generic counselling centre	48%	8%	1%	1.38
Psychotherapist in private practice	55%	17%	13%	2.27
Psychological counselling by general practitioner	44%	14%	11%	2.27
Psychological counselling by consultant	51%	17%	13%	2.31
Self-help group	64%	33%	10%	1.57
Pastoral services	50%	12%	4%	2.00
Any service				
Any psychological service	91%	68%	55%	n.a
Any social service	86%	65%	51%	n.a

About half of the patients (48%) reported that psycho-oncological consultations in the hospital had been *offered* to them, while 55% received the offer of consultations from a social worker. Similar proportions of patients were informed about services in the rehabilitation clinic. Services in the outpatient setting were offered less frequently—with self-help groups being the highest in ranking (33%) and generic counselling centres the lowest (8%).

*Uptake* of support in the hospital was highest for social services (41%) and lowest for pastoral care (11%). It was quite high in the rehabilitation clinic (of 242 patients who stayed at a rehabilitation clinic, 54% received an individual consultation by a psychologist) whereas in the outpatient setting only 9% received a consultation in cancer counselling centre, 13% went to a psychotherapist, 11% received psychological support from general practitioners, and 13% from oncology consultants.

The service offered and used most frequently was an individual consultation by a psychologist in the rehabilitation clinic.

*Psychological services of any form* (in the hospital, rehabilitation clinic, cancer counselling centre or private psychotherapy practice) was known by 91%, offered to 68%, and used by 55%. *Any form of social services* (in the hospital, rehabilitation clinic, or cancer counselling centre) was known by 86%, offered to 65%, and used by 51%.

### Reasons for non-use

Those who did not use services said most frequently this was because they did not need it (Table 3). The second most frequent reason for non-use of psychological services was disbelief in its effect, and for non-use of social services it was lack of knowledge that this existed.

**Table 3:** Reasons for not using psychosocial services among non-users

	Non-use of psychological services (*n* = 206)	Non-use of social services (*n* = 222)
I do not need such services	75%	69%
I do not believe it would help me	11%	6%
Other reasons	9%	4%
I eschew using such services	4%	2%
I did not know that this exists	4%	8%
I do not know whom to ask	2%	2%
It is too far for me	2%	0%

Other reasons mentioned in free texts explaining why a participant had ticked the category “other” were, for example, that she needed to care for her sick husband and did not have the time for herself or she had private insurance and feared she would not be reimbursed. Others thought that psychologists would only prescribe medication and this would make the situation worse.

### Perceived helpfulness

Overall, the users of psychosocial services rated it as helpful (Table 2). The highest scores in this aspect were given for social services at the hospital, psychological counselling by consultants in private practice, by general practitioners, and by psychotherapists in private practice. The lowest scores were given to group sessions with psychologists in rehabilitation clinics, to self-help groups, and to generic counselling centres.

### Distress and financial problems

At the time of cancer diagnosis, 29% of the patients reported mild, 9% moderate, and 2% moderately severe or severe distress. Five years later, this proportion was similar, with slightly more patients reporting moderate or severe distress (27% mild, 13% moderate, and 5% moderately severe or severe).

**Fig. 1:**
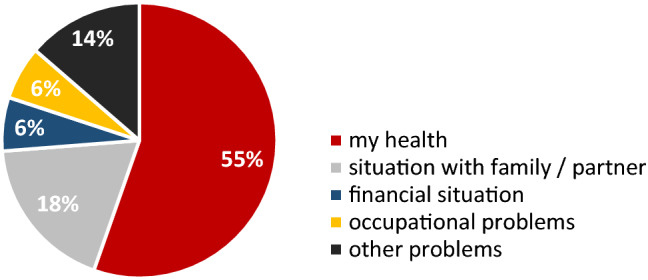
Distressing areas of life for breast cancer survivors

The most distressing aspects of life mentioned at *t*4 were one’s own health and the situation with family/a partner (Fig. 1). Other distressing problems mentioned were, for example, the health (or death) of relatives and friends, not being able to conceive, fatigue, tingling in hands and feet, or conflicts with tenants.

Financial problems above the threshold of clinical relevance were experienced by 21% at t1 and 33% at t4.

### Predictors of awareness, offer, and uptake

Different groups of patients were differently aware of the existence of social services. Women ≥ 65 years were less likely to know about it (odds ratio (OR) 0.2, *p* < 0.01) as well as women at increased risk (OR 0.4, *p* = 0.04). These services were also offered more frequently to and used by younger women (OR for older women 0.4, *p* < 0.01 and 0.3, *p* < 0.01, respectively). There was no evidence that patients with financial problems were offered or used social services more often than patients without financial problems (Table 4).

**Table 4:** Association of socio-demographic and clinical data as well as financial burden with awareness, offer, and use of social services

	Social services								
	Awareness			Offer			Use		
	OR	*P*	95% CI	OR	*P*	95% CI	OR	*P*	95% CI
Age at surgery									
< 65 years	Reference								
≥ 65 years	0.2	< *0.01*	(0.1–0.4)	0.4	< *0.01*	(0.3–0.6)	0.3	< *0.01*	(0.2–0.4)
Education									
< 10 years	Reference								
≥ 10 years	1.1	*0.76*	(0.7–1.6)	1.2	*0.31*	(0.9–1.6)	1.2	*0.28*	(0.9–1.6)
Income per person per month									
≥ 1500 €	Reference								
1000 to 1499 €	1.0	*0.98*	(0.4–2.2)	1.3	*0.38*	(0.7–2.3)	1.0	*0.90*	(0.6–1.8)
< 999 €	1.3	*0.52*	(0.5–3.3)	1.1	*0.79*	(0.6–2.0)	1.0	*0.97*	(0.5–1.9)
Unknown	0.9	*0.88*	(0.5–1.9)	0.9	*0.84*	(0.6–1.6)	1.2	*0.43*	(0.7–2.0)
Migration status									
No immigration history	Reference								
Immigrant	0.6	*0.14*	(0.3–1.2)	0.6	*0.09*	(0.3–1.1)	1.0	*0.89*	(0.5–1.7)
Risk (St. Gallen)									
Low	Reference								
Intermediate	0.2	*0.04*	(0.0–0.9)	0.5	*0.06*	(0.2–1.0)	0.7	*0.27*	(0.3–1.4)
High	0.2	*0.04*	(0.0–0.9)	0.6	*0.23*	(0.2–1.4)	0.7	*0.43*	(0.3–1.6)
Financial problems									
Below threshold	Reference								
Above threshold	1.4	*0.48*	(0.6–3.2)	1.1	*0.84*	(0.6–1.8)	1.3	*0.25*	(0.8–2.2)
Unknown	0.8	*0.62*	(0.3–1.9)	1.1	*0.74*	(0.6–2.1)	1.2	*0.53*	(0.7–2.2)

Notes: Displayed are the results of multivariate regression analysis, i.e. the effect estimates reflect effects while controlling for the other variables in the model

*P* = *P*-value

Psychological services were less often known about by women ≥ 65 years (OR 0.2, *p* < 0.01). There was no evidence that any other of the variables tested were associated with being aware of these services. Psychological services were less frequently offered to older women (OR 0.5, *p* < 0.01), and they also received them less frequently (OR 0.4, *p* < 0.01). These services were offered more often to and used by patients with increased distress (OR 2.0 for mild distress and 2.4 for moderate to severe distress; Table 5).

**Table 5:** Association of socio-demographic and clinical data as well as distress with awareness, offer, and use of psychological services

	Psychological services								
	Awareness			Offer			Use		
	OR	*P*	95% CI	OR	*P*	95% CI	OR	*P*	95% CI
Age at surgery									
< 65 years	Reference								
≥ 65 years	0.2	< *0.01*	(0.1–0.4)	0.5	< *0.01*	(0.3–0.7)	0.4	< *0.01*	(0.2–0.6)
Education									
< 10 years	Reference								
≥ 10 years	0.9	*0.42*	(0.6–1.2)	1.0	*0.91*	(0.8–1.4)	1.0	*0.74*	(0.8–1.4)
Income per person per month									
≥ 1500 €	Reference								
1000 to 1499 €	0.9	*0.89*	(0.4–2.4)	1.0	*0.96*	(0.6–1.8)	1.0	*0.90*	(0.6–1.7)
< 999 €	1.0	*0.99*	(0.4–2.8)	0.6	*0.09*	(0.3–1.1)	1.4	*0.30*	(0.7–2.6)
Unknown	1.1	*0.78*	(0.5–2.8)	0.5	*0.02*	(0.3–0.9)	0.8	*0.49*	(0.5–1.4)
Migration status									
No immigration history	Reference								
Immigrant	0.6	*0.21*	(0.2–1.4)	0.7	*0.24*	(0.4–1.3)	0.8	*0.42*	(0.4–1.4)
Risk (St. Gallen)									
Low	Reference								
Intermediate	0.4	*0.26*	(0.1–1.9)	1.0	*0.92*	(0.5–2.0)	1.8	*0.11*	(0.9–3.7)
High	0.3	*0.16*	(0.1–1.6)	1.0	*1.00*	(0.4–2.4)	1.2	*0.65*	(0.5–2.8)
Distress at time of surgery									
None	Reference								
Mild	2.0	*0.13*	(0.8–4.8)	1.6	*0.06*	(1.0–2.6)	2.0	< *0.01*	(1.3–3.2)
Moderate to severe	1.4	*0.61*	(0.4–5.1)	1.8	*0.11*	(0.9–3.7)	2.4	*0.01*	(1.2–4.8)
Unknown	1.2	*0.86*	(0.1–10.1)	1.3	*0.66*	(0.4–3.5)	0.9	*0.78*	(0.3–2.3)

Notes: Displayed are the results of multivariate regression analysis, i.e. the effect estimates reflect effects while controlling for the other variables in the model.

*P* = *P*-value

### How are the various services related to each other?

For the *offer* of services, there were four components with an Eigenvalue > 1. The first component was characterised by a relatively equal offer of all services. The second component was characterised by frequent offer of services in the rehabilitation clinic but few offers in the outpatient setting. The third component described women who had received very few service offers overall except social and pastoral services in the hospital as well as counselling by doctors in the outpatient setting. The fourth group had been offered the services in the hospital, especially pastoral care, and support by cancer counselling centres but rarely any form of psychological services and consultations with doctors in private practices (Table 1 in supplement).

The *use* of services had five components with an Eigenvalue > 1. The first component comprised women that used most types of services equally often. The second component is characterised by little use of services in the hospital and in the rehabilitation clinic but with high use of counselling by the oncologist in private practice, the general practitioner, and pastoral care. The third component groups women who rarely use pastoral care both in the hospital and in the outpatient setting but use all types of services in the rehabilitation clinic and counselling by doctors in private practice. The fourth component describes women who receive psychosocial support nearly exclusively from their general practitioner and occasionally from their oncologist but rarely from other sources. The fifth component clusters around use of pastoral services in the hospital and self-help groups with little use of support from physicians or psychologists (Table 2 in supplement).

## Discussion

The objective of this study was to define the proportions of breast cancer survivors who are aware of, have been offered, and have received professional and basic psychosocial support services. We distinguished these two forms of care because patients want and need both [34, 35]. We were also interested to further analyse who receives offers of such services and whether this is related to the actual need (distress, financial problems) and/or socio-economic or clinical variables independent of the need. Another question was how helpful the care received was from the perspective of the patients.

We found that a high proportion of breast cancer survivors are aware of the various services, and about half of them also used at least one psychological service and one social service. This could be due to the fact that all of them had been treated in hospitals certified by the German Cancer Society. To get such a certificate, it is a requirement that all patients are informed about psychosocial services [4]. In these certified centres, on average, 75% of all patients receive social services and 50% psychological services, according to the documentation of the centres. Breast cancer centres offer psycho-oncological consultation and social services even more frequently (70% and 88%, respectively) than other organ centres [4]. However, these are the percentages reported by the centres for the purpose of certification. From the perspective of the patients whom we asked in our study, the percentage of care actually received is obviously lower.

Indeed, it has been shown in a clinical trial with breast cancer patients that providing information by giving out flyers increased the awareness of psychosocial services; it did, however, not increase the use of these [36]. This implies that handing out flyers alone is not enough. Research shows that patients may need a more active recommendation to eventually go and visit psychosocial services, especially when they are ambivalent and highly distressed [37, 38].

Despite the screening policies in certified centres, older women in our study were less likely to be informed about and to receive offers for psychosocial services. This is concerning because they are also in need of it [39–41]. Possible explanations for this pattern are that health care professionals might think that younger patients are more interested in such support or they underestimate the need of elderly women for professional support. As Adler and Page rightfully underline [42], this is especially unfortunate as elderly women are more likely than younger ones to suffer from additional detrimental effects of other chronic conditions.

Reassuring to see is that patients with elevated distress also more frequently receive psychological services. This is important because the aim of optimal psychosocial care is not to give it to as many as possible but to those in need for it. Valdes-Stauber et al. also found that cancer patients with co-morbid mental health conditions receive more psychological consultations than those without [43]. Stepped care approaches can help to triage limited resources to those in highest need [44, 45]. Improvement in psychosocial care for cancer patients was observed in the United States by comparing National Health Interview Survey data between 2005 and 2010 and thereby showing that use of mental health services increased while the percentage of survivors with high levels of distress decreased [46]. Overall, this is reassuring because it shows that health policies indeed can improve psychosocial care over time.

For social services, this pattern was not as clear regarding financial problems. However, social services are of course offered not only for financial difficulties but also for other socio-economic and social needs [47].

Another interesting point is that the services best known were located in rehabilitation clinics but these services were considered not as helpful by the users than other services. It is possible that this is related to (a) the short duration of stays in the clinic with only limited time for consultations and (b) to the fact that everybody receives such offers, independent of their own wishes [9, 48, 49]. While this procedure has its value in making patients familiar with services, it is probably inherent that the helpfulness is evaluated less favourably. This is in line with findings from a meta-analysis [50] showing that patients identified as distressed via screening were less likely to use psychosocial services than unselected patients. These data should make us aware of the fact that structured screening and intervention programs also have their disadvantages [51]. There is no “one size fits all” in psycho-oncology. We must stay alert and in contact with our patients in order to be able to identify the ones in need and to offer them the support they require. In this respect, it becomes clear why what we called “basic psychosocial support” is indispensable. The doctors and nurses who work daily with the patients are part of the multi-disciplinary psychosocial team and can offer what a screening questionnaire cannot-listening “between the lines”, perceive what the patient expresses but cannot say, etc. Of course, what they need to be able to offer this is enough time, communication competence, and the ability for self-reflection [52, 53].

The service used least often and found least helpful was generic counselling centres. It is likely that this is due to the fact that patients need care providers who are familiar with cancer and its treatment [54].

Most helpful from the perspective of the survivors were social services in the hospital as well as individual consultations with consultants and with psychotherapists in private practices. It is also known from other studies that cancer survivors desire individual counselling by professionals the most in contrast especially to anti-depressants or other psychiatric medication [55], but medication is what they receive the most [56]. It seems that this is a specific problem in the US, where 44% of patients with breast cancer received psychotropic medication, compared to only 18% in Italy [21].

Self-help groups were well-known but not often used by our study participants (only 10%). This is in line with a study from Australia in patients with primary brain tumours, where only 25% were aware about and 13% had used self-help groups [57]. In other disease groups, these numbers have been found to be even smaller: an international survey by the Thyroid Cancer Association revealed that only 16% of the participants had been advised to visit a self-help group [58] and 4% of small cell lung cancer patients reported to have used it [18]. It seems that patients prefer to turn to professional help instead, maybe because they have misconceptions about what self-help groups can offer.

The findings of this study should be interpreted in the light of its limitations. Most notably, not all women who participated at baseline did so again 5 years later. This could have led to a bias by selecting those who are more motivated. If motivation to participate in a study and motivation so seek psychosocial help are related, which is not unlikely, our results overestimate the percentage of women who sought or used psychosocial services. Another limitation is that we did not further specify from whom help was received (e.g., the psychiatrist in the private practice, psychiatrist in the hospital, community nurse, etc.). This decision was taken because the questionnaire was already quite complex, and we wanted to keep a balance between comprehensiveness and burden for the patients.

In conclusion, we found that a high proportion of patients (but not all) are aware of psychosocial services, and those with a high need for such services indeed receive them more often. However, older women are less likely to be aware of and receive offers for psychosocial services independent of their distress level. This should be improved in the future. Services rated most helpful were social services in the hospital, psychological counselling by a consultant or a general practitioner, and psychotherapy in community-based practices, underlining the importance of multi-disciplinary approaches in patient care.

## Supplementary Information

Below is the link to the electronic supplementary material.

Supplementary file1 (DOCX 18 KB)

## Data Availability

The datasets generated and analysed during the current study are available from the corresponding author on reasonable request.
