# Supplementary material for: Awareness, offer, and use of psychosocial services by breast cancer survivors in Germany: a prospective multi-centre study
Source: Arch Gynecol Obstet. 2022 Jul 15;307(3):945–56. doi: 10.1007/s00404-022-06665-3 (PMC9984346; doi:10.1007/s00404-022-06665-3)
Supplement: Supplementary file 1 — Supplementary file1 (DOCX 18 KB) [file 404_2022_6665_MOESM1_ESM.docx]

Supplementary Table 1 (online only): Interrelations of which services were *offered*

|  |  | Comp 1 | Comp 2 | Comp 3 | Comp 4 |
| --- | --- | --- | --- | --- | --- |
| Hospital | Psychological consultation-liaison services | 0.29 | 0.26 | -0.02 | 0.28 |
|  | Social services | 0.18 | 0.31 | 0.42 | 0.27 |
|  | Pastoral services | 0.23 | 0.11 | 0.35 | **0.46** |
| Inpatient rehabilitation clinic | Consultation with psychologist | 0.22 | 0.32 | -0.17 | -0.25 |
|  | Group sessions with psychologist | 0.21 | **0.43** | -0.12 | -0.16 |
|  | Social services | 0.28 | **0.37** | -0.02 | -0.26 |
| Outpatient setting | Cancer Counselling Centre: Psychological services | 0.31 | -0.12 | -0.39 | 0.26 |
|  | Cancer Counselling Centre: Social services | 0.30 | -0.21 | -0.25 | 0.32 |
|  | Generic Counselling Centre | 0.24 | -0.36 | -0.18 | -0.03 |
|  | Psychotherapist in private practice | 0.31 | -0.08 | -0.11 | -0.40 |
|  | Psychological counselling by general practitioner | 0.26 | -0.27 | 0.37 | -0.27 |
|  | Psychological counselling by consultant | 0.27 | -0.17 | 0.43 | -0.27 |
|  | Self-help group | 0.30 | 0.00 | -0.24 | 0.01 |
|  | Pastoral services | 0.29 | -0.33 | 0.15 | 0.08 |

| *Notes* |  | Comp=Component |
| --- | --- | --- |
|  |  |  |
|  |  |  |

Supplementary Table 2 (online only): Interrelations of which services were *used*

|  |  | Comp 1 | Comp 2 | Comp 3 | Comp 4 | Comp 5 |
| --- | --- | --- | --- | --- | --- | --- |
| Hospital | Psychological consultation-liaison services | 0.34 | -0.28 | -0.10 | -0.17 | -0.06 |
|  | Social services | 0.26 | -0.33 | 0.15 | 0.03 | 0.17 |
|  | Pastoral services | 0.22 | 0.27 | -0.35 | 0.21 | **0.43** |
| Inpatient  rehabilitation clinic | Consultation with psychologist | 0.27 | 0.09 | 0.25 | -0.38 | 0.18 |
|  | Group sessions with psychologist | 0.19 | 0.18 | 0.34 | -0.31 | -0.12 |
|  | Social services | 0.32 | 0.04 | 0.30 | -0.14 | -0.03 |
| Outpatient setting | Cancer Counselling Centre: Psychological services | 0.35 | -0.32 | -0.06 | 0.17 | 0.05 |
|  | Cancer Counselling Centre: Social services | 0.26 | -0.45 | -0.09 | 0.22 | 0.21 |
|  | Generic Counselling Centre | 0.24 | 0.12 | -0.25 | -0.08 | -0.63 |
|  | Psychotherapist in private practice | 0.36 | -0.01 | -0.05 | 0.00 | -0.33 |
|  | Psychological counselling by general practitioner | 0.16 | 0.28 | 0.31 | **0.59** | -0.06 |
|  | Psychological counselling by consultant | 0.26 | **0.34** | 0.26 | 0.33 | 0.01 |
|  | Self-help group | 0.20 | 0.32 | -0.11 | -0.35 | **0.42** |
|  | Pastoral services | 0.22 | **0.27** | -0.58 | 0.00 | -0.05 |

| *Notes* |  | Comp=Component |
| --- | --- | --- |
|  |  |  |
|  |  |  |
